# Mapping and morphometric analysis of synapses and spines on fusiform cells in the dorsal cochlear nucleus

**DOI:** 10.3389/fnsys.2014.00167

**Published:** 2014-09-23

**Authors:** Rony H. Salloum, Guoyou Chen, Liliya Velet, Nauman F. Manzoor, Rachel Elkin, Grahame J. Kidd, John Coughlin, Christopher Yurosko, Stephanie Bou-Anak, Shirin Azadi, Stephanie Gohlsch, Harold Schneider, James A. Kaltenbach

**Affiliations:** Department of Neurosciences, Lerner Research Institute and Head and Neck Institute, Cleveland ClinicCleveland, OH, USA

**Keywords:** serial block face scanning electron microscopy, dorsal cochlear nucleus, fusiform cells, dendritic spine volumes, parallel fibers, synaptic vesicles, active zone, postsynaptic density

## Abstract

Fusiform cells are the main integrative units of the mammalian dorsal cochlear nucleus (DCN), collecting and processing inputs from auditory and other sources before transmitting information to higher levels of the auditory system. Despite much previous work describing these cells and the sources and pharmacological identity of their synaptic inputs, information on the three-dimensional organization and utltrastructure of synapses on these cells is currently very limited. This information is essential since an understanding of synaptic plasticity and remodeling and pathologies underlying disease states and hearing disorders must begin with knowledge of the normal characteristics of synapses on these cells, particularly those features that determine the strength of their influence on the various compartments of the cell. Here, we employed serial block face scanning electron microscopy (SBFSEM) followed by 3D reconstructions to map and quantitatively characterize synaptic features on DCN fusiform cells. Our results reveal a relative sparseness of synapses on the somata of fusiform cells but a dense distribution of synapses on apical and basal dendrites. Synapses on apical dendrites were smaller and more numerous than on basal dendrites. The vast majority of axosomatic terminals were found to be linked to other terminals connected by the same axon or different branches of the same axon, suggesting a high degree of divergent input to fusiform cells. The size of terminals was correlated with the number of mitochondria and with the number of active zones, which was highly correlated with the number of postsynaptic densities, suggesting that larger terminals exert more powerful influence on the cell than smaller terminals. These size differences suggest that the input to basal dendrites, most likely those from the auditory nerve, provide the most powerful sources of input to fusiform cells, while those to apical dendrites (e.g., parallel fiber) are weaker but more numerous.

## Introduction

The dorsal cochlear nucleus (DCN) is a multimodal processing station that lies at the junction of the auditory nerve and brainstem medulla (Shore, 2005). This nucleus consists of three layers, including an outer molecular layer, a middle fusiform cell layer and a deep polymorph layer (Osen and Mugnaini, 1981). The main integrative units of the DCN are the fusiform cells, which extend their apical dendrites into the molecular layer and their basal dendrites into the deep layer (Brawer et al., 1974). Ultrastructural and immunohistochemical studies have yielded a wealth of information on the stratification of synaptic input types to fusiform cells. The spine-rich apical dendrites receive excitatory inputs primarily from parallel fibers (Oliver et al., 1983; Blackstad et al., 1984; Wouterlood and Mugnaini, 1984; Mugnaini, 1985; Hirsch and Oertel, 1988; Manis, 1989; Berrebi and Mugnaini, 1991; Osen et al., 1995; Waller et al., 1996; Rubio and Wenthold, 1999; Rubio and Juiz, 2004). The receptors for these inputs are glutamatergic and include AMPA, kainite-sensitive and NMDA subtypes (Oliver et al., 1983; Juiz et al., 1993; Godfrey et al., 1994; Osen et al., 1995; Waller et al., 1996). Most other inputs to the apical dendrites are inhibitory in function and come from nearby cartwheel and stellate cells, and from midbrain auditory nuclei (Mugnaini, 1985; Wenthold et al., 1986; Adams and Mugnaini, 1987; Berrebi and Mugnaini, 1991; Rubio and Juiz, 2004). Inputs to the cell body are thought to be mostly inhibitory and come from neighboring cartwheel cells, vertical cells, D-stellate cells in the ventral cochlear nucleus and descending pathways from midbrain auditory nuclei (Berrebi and Mugnaini, 1991; Zhang and Oertel, 1994; Rhode, 1999; Davis and Young, 2000; Rubio and Juiz, 2004). The basal dendrites have few spines and receive excitatory inputs mainly from the auditory nerve (Cohen et al., 1972; Manis and Brownell, 1983; Oliver et al., 1983; Smith and Rhode, 1985; Ryugo and May, 1993; Rubio and Wenthold, 1997), but possibly also from T-stellate cells in the ventral cochlear nucleus and cells from other sources (Oertel et al., 1990; Rubio and Juiz, 2004). Inhibitory inputs to the basal dendrites are thought to come from sources similar to those contacting the soma surface.

Thus far, the synaptologies of fusiform cells have been described almost exclusively on the basis of two-dimensional imaging of single or “short stacks” of adjacent thin sections spanning a few micrometers. While this approach has provided much information about the sources and types of synaptic inputs to fusiform cells, the quantitative aspects of these features have received relatively little attention. Such issues as how synapses are spatially mapped over the soma and dendritic spines, and the 3D characteristics of features that determine synaptic strengths, such as spine and terminal volumes, number of synapses and their active zones, mitochondria and postsynaptic densities (PSDs), have not yet been characterized. Here, we employed serial block face scanning electron microscopy (SBFSEM) followed by 3D reconstructions to map and characterize these features on the apical and basal dendrites and somata of DCN fusiform cells.

## Materials and methods

### Animal subjects

Animals used in this study were adult (80–82 days of age) male Syrian golden hamsters. The choice of the hamster is based on previous publications demonstrating similar anatomical and physiological cell types and overall cytoarchitectural and tonotopic organization patterns to those of other rodent species in common use in the laboratory (Schweitzer and Cant, 1985; Schweitzer, 1990, 1991; Kaltenbach and Lazor, 1991; Finlayson and Kaltenbach, 2009) as well as its suitability for and relevance to studies of neural plasticity in states of abnormal hearing (Kaltenbach et al., 2004; Manzoor et al., 2013). These were obtained from Charles River and were housed in an animal vivarium on a 12 h:12 h light/dark cycle for 16–20 days before use. The protocol used in this study was approved by the animal investigation committee (IACUC) of the Cleveland Clinic, and the animals were cared for in accordance with the NIH Guidelines for the Care and Use of Animals in Research.

### Brain tissue fixation

Animals were anesthetized with ketamine/xylazine (117 mg/kg–18 mg/kg), then perfused transcardially with 500 ml of 4% paraformaldehye, 2% glutaraldehyde and 0.1 M sodium cacodylate. The brain was carefully removed from the cranium and post-fixed in the same fixative for 24 h. The fixed brain was dissected under a light microscope into a block of tissue that contained the left DCN, medulla, pons and left IC. Inclusion of all these structures gave each sample a distinct shape, which was hand drawn to aid in localizing the DCN later after staining. The tissue was stained with heavy metals in the following solutions alternated either with water or cacodylate washes: 0.1% tannic acid for 60 min followed by ferrocyanide-reduced 2% osmium tetroxide (OsO_4_) for 60 min, 1% thiocarbohydrazide (TCH) solution at 60°C for 20 min followed by 2% OsO_4_ solution for 30 min, then in 1% uranyl acetate solution overnight at 4°C, and lastly, in 20 mM lead aspartate solution at 60°C for 30 min. The tissue was subsequently dehydrated in increasing concentrations of ethanol, then in 100% acetone. Tissue was embedded first in 50:50 mixtures of acetone and EPON overnight at room temperature, then in fresh EPON (100%) in a mold for 48 h at 60°C under a vacuum.

### Block preparation

Under a dissecting microscope, each embedded tissue block was placed on a chuck with the dorsal surface of the DCN facing upwards. The block was then cut in the transverse plane at a level approximately half-way between the rostral and caudal borders of the DCN. The block was placed inside the column of a Zeiss Sigma VP scanning electron microscope, equipped with a Gatan 3View microtome, and examined at 200×, to confirm the orientation of the DCN. At this magnification, the left DCN appeared crescentic and straddled the dorsolateral surface of the medulla just above the restiform body. The region of interest was defined as a rectangular area spanning the three layers of the DCN at a position approximately 30% of the distance from the medial to the lateral borders of the DCN. After an image of the DCN was obtained, the block was then removed from the microscope and further trimmed into a cube that encompassed the region of interest and measured roughly 0.5 mm × 0.5 mm.

### Slicing and image acquisition

The final trimmed block was mounted onto the microtome stage with the transverse face of the DCN facing upwards. Serial sections were cut at a thickness of 75–80 nm. The magnification was then set at 4000×, and the electron beam set at 2.0 kV using an aperture of 30 μm. The block face was then imaged at a resolution of 10–11 nm/voxel using a dwell time of 1.0 μs/voxel. For each section, images were captured sequentially in six adjacent but overlapping square fields, each measuring 75–80 μm/side. Together, the overlapping field spanned the molecular, fusiform cell and outermost part of the deep layers of the DCN. The six fields were imaged in a total of 550–650 sections. The end result was a set of six image stacks that covered an area measuring 225 μm × 240 μm and spanned a rostrocaudal thickness of 40 μm.

### Segmentation

After image acquisition, the six stacks were stitched together using TrakEM2 software (ImageJ/Fiji) into a montage, within which the outlines of fusiform cells and their dendrites and synapses were traced and color coded. The stitching process generally resulted in good alignment of slices between stacks. When misalignments were observed that were potentially problematic to the segmentation process, they were corrected manually by sliding the image to maximize matches of details between corresponding slices in adjacent stacks. To obtain insight into how synapses are spatially distributed over the soma and dendrites, we traced through the image stacks both of these features and all synaptic profiles bordering on their surfaces. We also separately traced the spines on dendrites and the presynaptic terminals impinging on the spines.

### 3D reconstruction

Following segmentation, 3D reconstruction of each neuron was accomplished by merging the traced features along the Z-axis of the image stack using TrakEM2 software. Rendering of surface features was performed using Blender software.

### Data analysis

To quantify synaptic terminals, each terminal in the reconstructed image was given its own I.D. number. Each terminal was then isolated from the most distal point of the axon where there was a clear distinction between axon and terminal (i.e., where the axon’s diameter did not change with distance from the terminal). Volumes of terminals and spines were computed using TrakEM2 software. Measures of mean terminal and spine volumes were performed for each synaptic compartment of the cell (apical dendrite, basal dendrite, soma). The volume of each identified spine was measured from the point where the spine met the dendritic shaft to its distal extremity. The total number of mitochondria inside each axonal terminal was counted by numbering each mitochondrion that was found not to be connected with a neighboring mitochondrion when traced in successive sections. All measures were averaged across neurons, yielding a group mean for each feature of interest.

## Results

**Figure 1:**
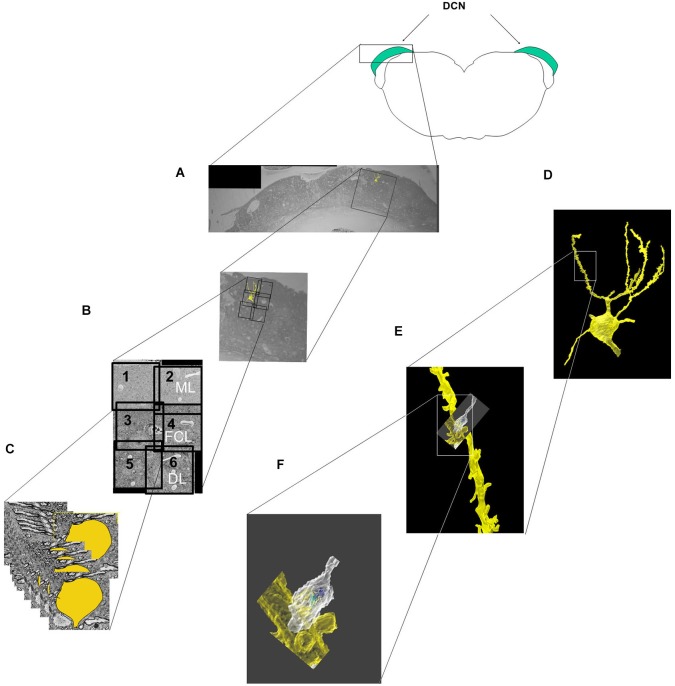
**Steps involved in the imaging, segmentation and reconstruction of fusiform cells and their synaptic features**. **(A)** A block containing the DCN was cut in the transverse (coronal) plane to show the location of the fields selected for imaging in relation to the different layers and medio-lateral extent of the DCN. The block was then trimmed further into a smaller cube, and 500–600 slices were cut and sequentially imaged in each of six subfields (labeled 1–6). **(B)** The six image stacks were then stitched together into a montage that spanned much of the dorsoventral thickness of the DCN and included the molecular layer (ML), the fusiform cell layer (FCL) and the outermost part of the deep layer (DL). **(C)** In the segmentation step, features of interest (cell somata, dendrites, spines and synapses) were separately traced and color-coded in each slice. **(D–F)** The features of interest were merged along the Z-axis of the image stacks, resulting in the 3D reconstruction of the cell **(D)**. A segment of an apical dendrite **(E)** and an axonal terminal contacting a spine **(F)** are shown at higher magnifications.

The locations of the six imaged subfields relative to the DCN borders are shown in Figure 1A. The five neurons analyzed in this study were obtained from two animals (two neurons in one animal, three from the other). The soma of each neuron was located in the fusiform cell layer (FCL), which corresponds to the area encompassed by subfields three and four in Figure 1B. After the soma and dendrites were traced through the image stack (Figure 1C), the tracings were merged to yield the 3D reconstruction of the cell surface, which was analyzed over its entirety at different levels of magnification (Figures 1D–F).

### Fusiform cell morphologies

**Figure 2:**
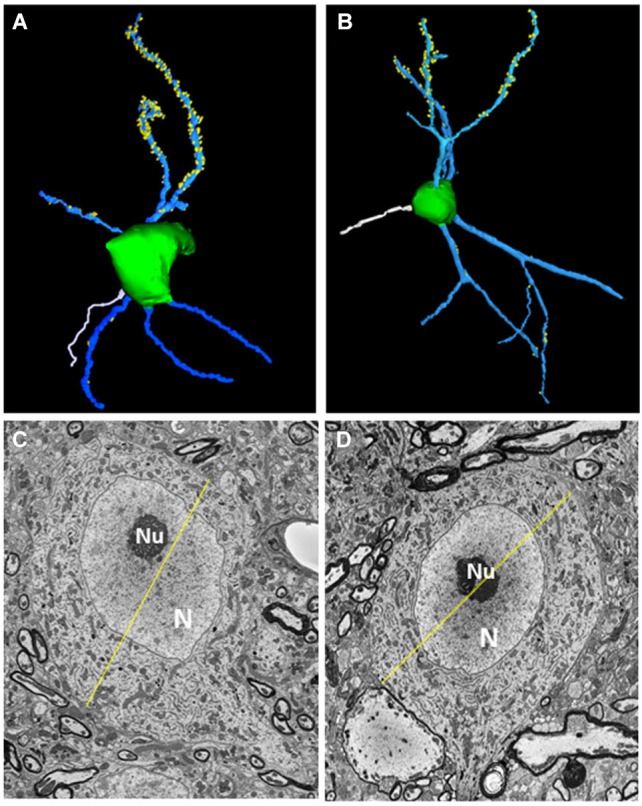
**Morphological features of fusiform cells**. **(A,B)** 3D-reconstructions of the cell somata (green) and selected apical dendrites (blue) containing spines (yellow). An axon (white) can be seen extending from the soma of each neuron. **(C,D)** Transverse sections through each fusiform cell shown in **(A,B)** at levels where they reached their largest diameters (yellow lines). The diameters were 26.38 μm for the fusiform cell shown on the left and 24.4 μm for the other cell. N: Nucleus, nu: nucleolus.

All five fusiform cells analyzed were among the largest cells in the fusiform cell layer. Each was elongated along one axis and extended dendrites apically into the molecular layer and basally into the deep layer (see examples in Figures 2A,B). The somata had maximal diameters averaging 24.83 ± 1.57 μm (mean ± S.D.) with a range of 22.7–26.4 μm (Figures 2C,D), consistent with their identity as fusiform cells, which have been found previously to have maximal mean diameters between 20 and 30 μm in the mouse (Ryugo and Willard, 1985) and rat (Rubio and Juiz, 2004) and between 20 and 35 μm in the cat (Kane, 1974). Their larger size distinguishes them from nearby cartwheel cells which are much smaller and range from 10 to 14 μm in mean diameter and rarely exceed 18 μm in maximal diameter (Wouterlood and Mugnaini, 1984). The primary branches of the apical and basal dendrites typically divided into secondary and tertiary branches. Spines were generally present on both apical and basal dendrites, but were most consistently and most densely distributed on the lengths of secondary and tertiary apical dendritic branches that reached into the middle and upper levels of the molecular layer. Fewer spines were present on basal dendrites or on primary apical dendritic segments present in the deepest third of the molecular layer. Each cell displayed an axon (shown in white in Figures 2A,B) which took origin from the soma and became myelinated within the first 26–34 μm from the soma. When the axons could be traced far enough to observe their trajectories, they were found to project laterally a short distance before descending ventrally toward the dorsal acoustic stria (i.e., the fiber tract that carries axons of fusiform cells on their way to the contralateral inferior colliculus). The cells showed a distinct nucleus that was either centrally or eccentrically located. In most sections the nucleus appeared round or oval (Figures 2C,D), but in some sections, the nucleus was deeply indented or divided into lobes, reflecting a complex shape.

### Total number of synaptic inputs to fusiform cells

We categorized a structure as a synaptic terminal if it showed a clearly established contact with the soma and/or its dendrites and could readily be distinguished from other elements of neuropil and glial cells based on size, shape and the axon-like characteristics of the connecting branch from which it extended. The total number of synaptic terminals to the various compartments of the cell is exemplified by fusiform cell FC3. This example was chosen because the apical and basal dendritic arbors were approximately similar in their number of branches and their approximate lengths. This cell received a total of 621 terminals, of which 312 were distributed to the apical dendrite, 251 were on the basal dendrite and 58 were on the soma. Of the synaptic inputs to the apical dendrite, 126 ended on spines while 186 ended on shafts. Of the inputs to the basal dendrite, 214 were on shafts and only 37 were on spines.

### Ultrastructural features of the neuropil surrounding fusiform cells

**Figure 3:**
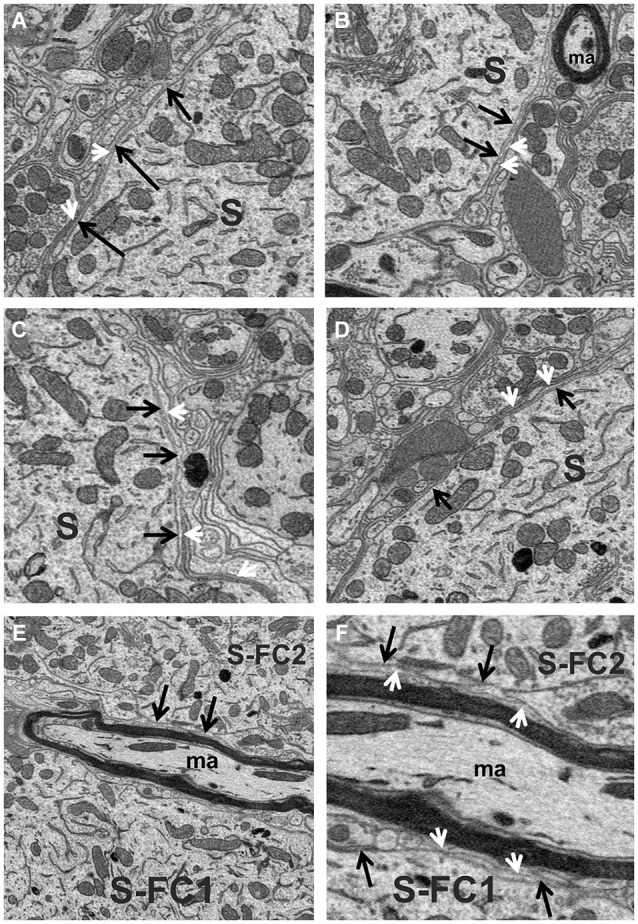
**(A–D)** High magnification view of the fusiform cell plasmalemma (black arrows) showing the thin astrocytic lining (white arrowheads) that ensheathed much of the soma (S) surface. **(E,F)** Different magnifications of the surfaces (black arrows) of two fusiform cell somata (S-FC1 and S-FC2) that were very closely associated. Note the band of myelinated axons (ma) that runs between these cells was separated from the somal surface by astrocytic processes (white arrowheads).

The space between fusiform cells was densely packed with a variety of elements, including dendrites, myelinated and unmyelinated axons, the somata of other neurons, blood vessels and neuroglia. The most common glial cell type observed was the astrocyte, whose thin processes typically extended long distances forming multiple layers of glial sheaths that partially enveloped the fusiform cell soma and filled much of the space between neighboring axo-somatic synapses; the multilayer characteristic of the sheath can be seen in the example of Figures 3A–F. Astrocytic processes were also found ensheathing the different segments of dendrites that were not contacted by synaptic terminals. These glial sheaths extended from their terminal-like swellings in contact with the fusiform cell soma or dendritic shaft. The sheaths from different astrocytes thus formed a more or less continuous lining around most of the surfaces of somata and dendrites not contacted by synapses, although other elements of neuropil, such as axons (both myelinated and unmyelinated) and other dendritic branches were also commonly seen contacting the surfaces of dendrites.

### Axo-somatic synapses on fusiform cells

**Figure 4:**
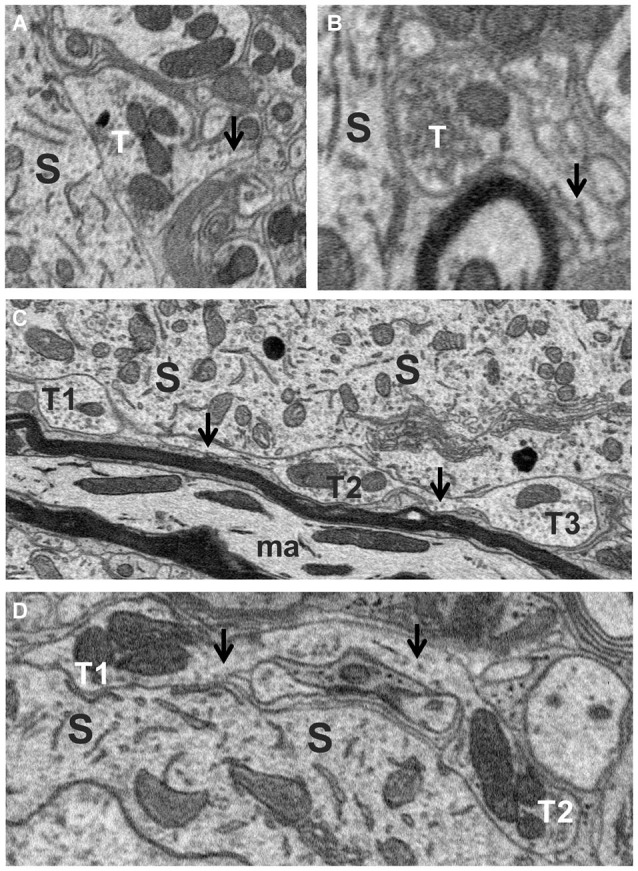
**Axo-somatic terminals on a fusiform cell**. **(A,B)** Examples of terminal boutons (T) in contact with a soma (S). Note the axons (arrows) of these terminals heading away from the terminal in the direction opposite the soma. **(C)** Examples of three *en passant* swellings (T1, T2 and T3) in contact with the soma (S) along the trajectory of an axon (arrows). **(D)** Two *en passant* swellings (T1, T2) belonging to the same axon (arrows) contacting the soma.

Axosomatic synapses were generally recognized by the presence of a soma-contacting membrane forming a rounded sac-like or elongated envelope budding off an axon and enclosing a distinctly dark staining population of neurotransmitter vesicles and numerous mitochondria. A total of 455 synaptic contacts were traced on the somata of the five fusiform cells studied. Just under half (47%) of these were terminal boutons (Figures 4A,B), while the remainder (53%) occurred as en passant swellings giving off two or more branches. Many profiles initially appearing as terminal boutons when examined in only a few adjacent sections were found to be boutons *en passant* when traced over their full thicknesses. Indeed, tracing their axons revealed a high degree of interconnectivity among synapses. When axons could be traced retrogradely or anterogradely from their synaptic contacts over short distances, different terminals or *en passant* swellings were often found to be linked by a common axon (T1–T3 in Figure 4C, T1 and T2 in Figure 4D). To ascertain how common these linkages were, we traced axons from a subset of axosomatic synapses. Out of 93 synapses, 82 could be traced successfully over distances of at least a few microns, and within this distance, the majority (49/82) were found to be linked to one or more other nearby synapses. The mean number of terminals linked by a common axon over this range was 3.02 (range 1–10). This suggests that the number of axons providing input to the soma may be much smaller than the number of contacting synapses.

**Figure 5:**
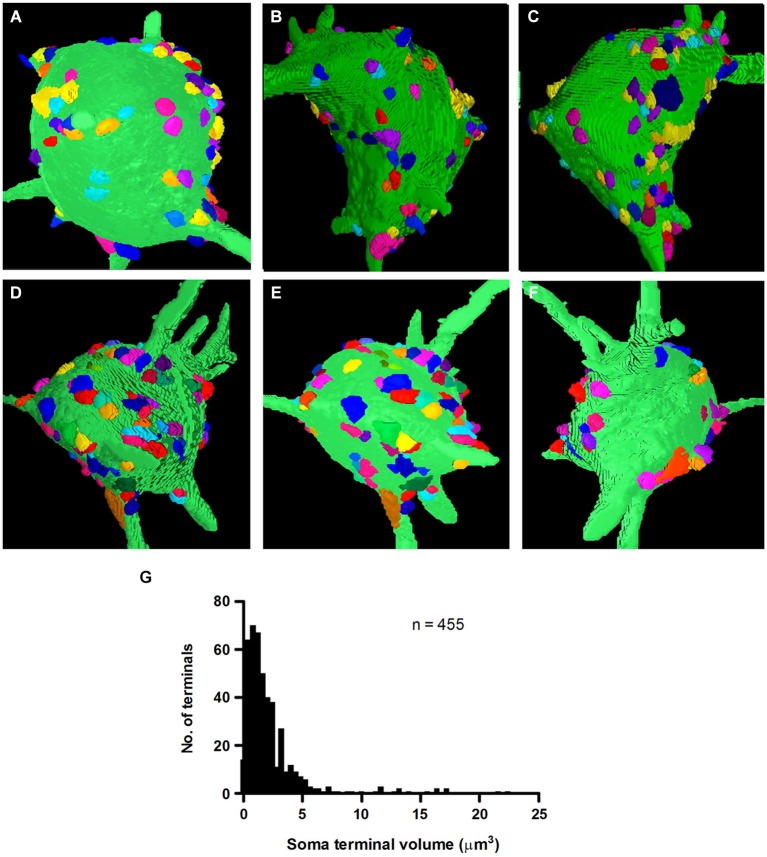
**3D-reconstructions of two fusiform cell somata, FC1 (A–C) and FC2 (D–F) observed from three angles of rotation to show the distribution of their inputting axo-somatic synapses**. Note the wide spacing between terminals. Each terminal is represented by a different color so that they can be distinguished even when they occur in aggregates. **(G)** Volume distribution histogram for presynaptic terminals on the somata of five fusiform cells.

Multi-angle views of two reconstructed fusiform cells along the Z-axis of our image stacks revealed the distribution of synapses on their somal surfaces (Figures 5A–F). The reconstructions show the large areas of somal surface that were unoccupied by synapses. The mean number of synapses of clear neuronal origin contacting fusiform cell somata was 91.0 ± 19.46 (*n* = 455 on the five cells), with a range of 58 to 109 (FC1-100, FC2-109, FC3-58, FC4-95, FC5-93). There was a broad distribution in the sizes of these synapses, excluding the non-terminal portion of the axon (Figure 5G). When all the terminals were pooled across the five cells, the mean volume was 2.51 ± 2.86 μm^3^ (mean ± SD), with a range of 0.1 to 22.4 μm^3^. The vast majority (94%) had volumes of less than 6 μm^3^, while a much smaller proportion (6%) had larger volumes reaching up to 22.4 μm^3^. The mean volumes of axo-somatic synapses per cell were very similar, varying only slightly from 2.32 ± 2.54 μm^3^ in FC2 to 2.76 ± 3.08 μm^3^ in FC6.

The ability to identify terminals belonging to the same axon provided an excellent opportunity to test whether terminals that were linked by a common axon were similar in size. Surprisingly, with close inspection of 29 sets of linked terminals, we found no evidence that small and large terminals originated from different axons. One of our larger sets of linked synapses, which contained 10 interconnected terminals, showed almost the same mean (2.96 μm^3^) and range (0.48–8.32 μm^3^) of terminal volumes, as the population of axosomatic terminals as a whole (Figure 5G). Moreover, the number of terminals below this mean value in this set was similar to the number of terminals above the mean. These observations indicate that terminal volume cannot by itself be used to categorize inputs from a single source. These size differences do nonetheless raise the possibility that synapses may differ significantly in the strength of their influence on the postsynaptic cell.

### Active zones and mitochondria of axo-somatic synapses

**Figure 6:**
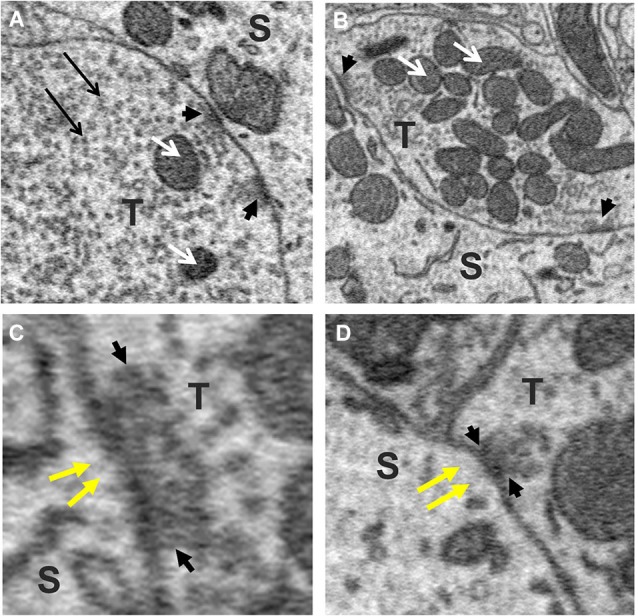
**Active zones of axo-somatic terminals on fusiform cells**. **(A,B)** Examples of terminals (T) containing multiple active zones (small black arrow-heads). White arrows point to mitochondria. **(C,D)** Higher magnification views of synaptic junctions containing active zones (cluster of vesicles between black arrowheads pointing towards each other). Note the absence of PSDs beneath the postsynaptic membrane (yellow arrows), indicating that these were symmetric synapses. The vesicles of the active zone appear amidst a larger (reserve) pool of vesicles that fill the rest of the terminal (long black arrows in **A**).

**Figure 7:**
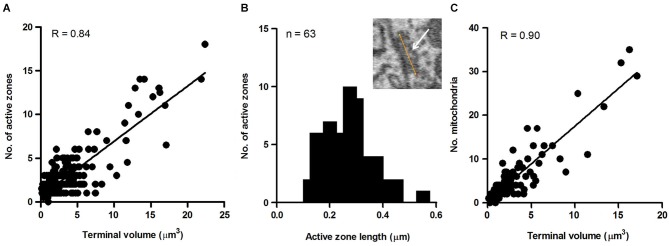
**Measures of axo-somatic terminals on fusiform cells**. **(A)** Relationship between the size of the axonal endings (*n* = 455) on fusiform cell somata and the number of active zones inside those endings. **(B)** Maximum length distribution of active zones measured in the terminals synapsing on somata. The maximum length refers to the distance spanned by the yellow line paralleling the active zone (white arrow) shown in the inset (*n* = 63). **(C)** Relationship between terminal volume and number of mitochondria. The data were taken from a random sample (*n* = 89) of axo-somatic terminals of three fusiform cells.

To explore this possibility further, we sought to determine whether the size of the terminal predicted the number of active zones they contained. This was motivated by previous studies reporting that the number of active zones correlates with quantal size and neurotransmitter release probability and therefore should provide a reasonable estimate of the relative strength of synapses (Schikorski and Stevens, 1997; Meyer et al., 2001). Active zones in our sample of axosomatic terminals were identified as areas within the synaptic ending where neurotransmitter vesicles were concentrated in clusters or bands flanking the pre-synaptic membrane where it contacted the soma. Active zones differed from the rest of the vesicle pool in which vesicles were more diffusely distributed and seemingly scattered more or less at random throughout the terminal. Surprisingly, a small percentage of terminals were completely devoid of active zones, suggesting that some terminals were inactive. The preponderance of axosomatic terminals examined (*n* = 435/455), however, possessed well defined active zones (Figures 6A–D, small black arrowheads), consistent with an active role of most terminals in neurotransmission. Moreover, there was considerable variation in the number of active zones from terminal to terminal, which allowed us to examine their relationship with terminal size. Most (92%) of the synaptic endings on the somata (418/455) were small (under 5 μm^3^) and had few (1–3) active zones. The largest terminals were more than 15 μm^3^ and had more than 10 active zones, with the maximum number being 17. A plot of the number of active zones vs. the size of the terminal revealed a strong correlation between the two quantities, with larger terminals showing higher numbers of active zones and fewer active zones in smaller terminals (*R* = 0.84, *P* = 0.0001; Figure 7A). Moreover, there was little difference between the sizes of active zones in terminals of different volumes. The average thickness of active zones was 350–380 nm in both large and small terminals, while the maximal length of active zones in different size profiles (the length between the arrow heads in Figures 6C,D) ranged from 0.13 to 0.55 μm with a mean of 0.28 ± 0.89 μm (mean ± SD) (Figure 7B). The number of mitochondria contained in the axonal terminal was also found to correlate strongly with the size of the terminal (Figure 7C). These findings serve to confirm the correlation between the volume of presynaptic terminals and the number of active zones they contain and suggest that larger terminals were more active than smaller terminals.

### Postsynaptic densities (PSDs) of axosomatic synapses

Most (73%) axo-somatic synapses lacked PSDs flanking the majority of their active zones (Figures 6C,D, yellow arrows). These are consistent with their identity as symmetric synapses (Gray type II). The remaining 27% showed well-developed or slightly tinted cytoplasm below most of their active zones. These features are consistent with their identity as asymmetric synapses (Gray type I). The fact that different synaptic terminals were found to be linked by a common axon on our cells allowed us to test whether all terminals associated with the same axon were characterized by similar types of synapses (type I or II). We examined 77 total synapses that were associated in 29 sets of linkages ranging from 2 to 10 synapses/set. Eighteen of these sets contained synapses that were either all symmetric or all asymmetric; the remaining 10 sets contained a mixture of symmetric and asymmetric synapses. Therefore, symmetry by itself cannot be used to categorize inputs from a single source.

### Dendritic spines

**Figure 8:**
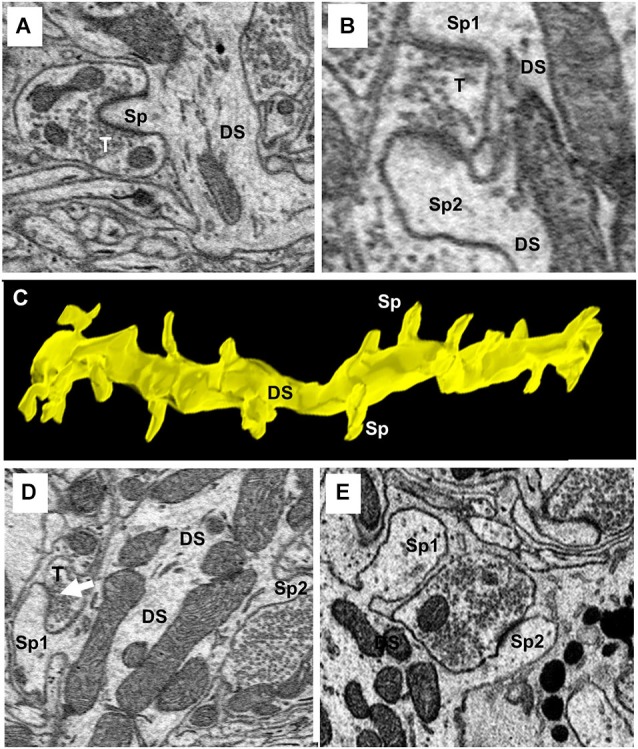
**Ultrastructure and reconstruction of apical dendritic spines**. **(A)** A single spine (Sp) completely surrounded by a single axo-spinous terminal (T). **(B)** An example of an axo-spinous terminal synapsing with two different spines (Sp1 and Sp2) of the same dendritic shaft (DS). **(C)** 3D-reconstruction of a segment of a secondary branch of an apical dendrite showing the stubby morphology of spines. DS, dendritic shaft. Sp, spine. **(D)** A mushroom spine (Sp1) is shown extending from the left side of the dendritic shaft (DS). **(E)** Two spines on the same dendrite showing the head and neck components of the spine. Note the terminal contacting Sp2 extended beyond the spine to contact the shaft.

**Figure 9:**
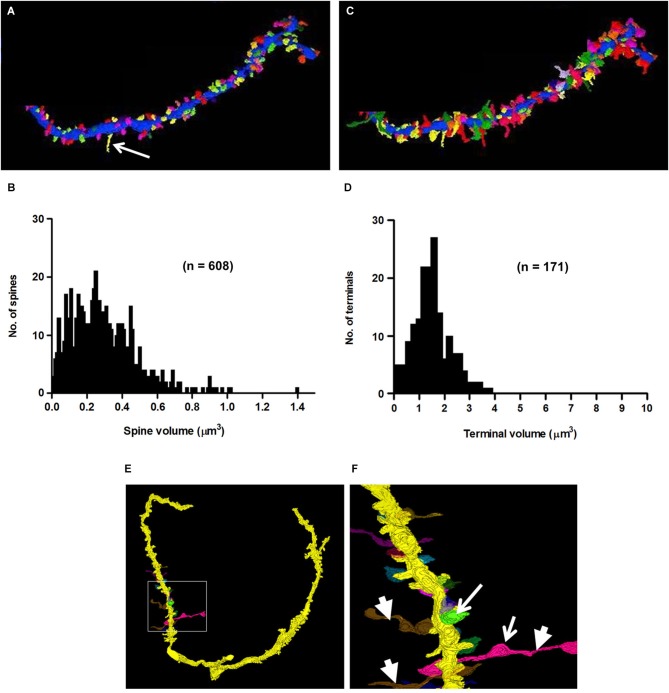
**3D-reconstructions and metrics of spines and axo-spinous terminals of a secondary branch of an apical dendrite**. **(A)** Cartography of spines, represented in different colors. Arrow points to a filopodium. **(B)** Histogram showing the probability distribution of spines of different volumes. **(C)** Reconstruction of synaptic terminals, (different colors) ending on spines shown in panel **A**. **(D)** Probability distribution of terminals of different sizes on dendritic spines (compare with Figure 5G). Note that the short stretches of axon beyond the terminal shown in panel **(C)** were not included in the measures of terminal volume (see Section Methods). **(E–F)** 3D-reconstructions of axons showing their *en passant* swellings (arrows) and horizontal trajectories (arrowheads) consistent with their identities as parallel fibers.

One of the characteristics that distinguishes fusiform cells from other cell types is the preponderance of spines on their apical dendrites while relatively few are present on their basal dendrites (Blackstad et al., 1984; Smith and Rhode, 1985). On the apical dendrites of our fusiform cells, spines were most densely distributed on secondary and tertiary branches of the apical dendrites, although, as can be seen in Figures 2A,B, there was much variation in the density from cell to cell, from branch to branch and from one segment of a given branch to another. The spine density, along the segments of apical dendrites where the population of spines was highest (typically, the segment in the middle and outer strata of the molecular layer), ranged between 1.02 and 1.76 spines/μm across branches with a mean density of 1.37 spines/μm. Cross sections through the apical dendrites usually showed poor differentiation of spine head and neck regions (Figures 8A,B). As shown by the 3D reconstruction in Figure 8C, the spines were mostly stubby; mushroom-shaped spines were occasionally observed, as in the example of Figure 8D (Sp1). Volume measurements of spines were based on the entire spine, irrespective of shape; filopodia, which were rarely observed (arrow in Figure 9A), were not included among the spines in these measurements. Apical dendritic spine volumes ranged from 0.01 to 1.40 μm^3^ (Figure 9B) and averaged 0.29 ± 0.19 μm^3^ (mean ± SD) (*n* = 738).

Spines on basal dendrites were generally much more sparsely distributed than those on apical dendrites. Indeed, most branches of the basal dendrites showed long stretches with only a few or a complete absence of spines. However, spine density varied greatly across branches and across segments of the same branch. For example, spine density on certain stretches of the proximal portion of the basal dendrite of one of our neurons reached 1.02 spines/μm, comparable to the spine density of secondary branches of the apical dendrites. Nonetheless, even in these segments with high spine density, spines on the basal dendrites were generally much smaller than those on the apical dendrites, averaging only 0.10 ± 0.08 μm^3^ (*n* = 65) or roughly one third the volume of spines on the apical dendrites. These findings suggest that spines on basal dendrites probably have an important function, but their role is likely to be considerably different from that of spines on the apical dendrites.

### Axo-dendritic synapses

Synaptic terminals were traced on the shafts and spines of all apical and basal dendrites of our cells. A total of 656 synapses, both shaft and spine, were traced on the apical dendrites. Of those synapses, 387 (59%) were shaft terminals, while 269 (41%) were spine terminals. Clearly defined synaptic profiles were found on 65% (173/269) of these spines, and spines contacted by more than one terminal were less commonly seen. The mean volume of shaft terminals on the apical dendrites was 2.46 ± 2.43 μm^3^ (mean ± SD) and varied little across cells, ranging from 1.38 μm^3^ in FC2 to 3.31 μm^3^ in FC7. The mean volume of spine terminals on the apical dendrites was 1.73 ± 1.74 μm^3^ (mean ± SD), but unlike those on shafts, varied greatly across cells (Figure 9D). For example, those on FC1 and FC2 had mean volumes of 1.63 ± 0.78 and 1.68 ± 0.82 μm^3^, respectively, while those on FC6 averaged 7.55 ± 3.5 μm^3^. These differences suggest that the inputs to fusiform cells are not identical across cells. Almost all of these terminals (160/173, 92%) contacted only a single spine as in Figure 8A; the remainder contacted 2–3 spines (Figure 8B). Most 101/173 (58%) of the contacts made with spines were terminal boutons. The remaining 42% of contacts were *en passant* swellings (72/173). In addition, 61 of the terminals contacting spines extended beyond the borders of the spines to contact the dendritic shaft between the spines (Figure 8E). This characteristic was most apparent on spines of the portion of the apical dendrites in the deeper part of the molecular layer and on spines of the basal dendrites where most of the very large terminals were found. Reconstructions of a selected set of terminals are shown in Figures 9C,E,F. When possible, the terminals were traced retrogradely to include a short stretch of axon. These were found to be unmyelinated, to contain multiple *en passant* swellings, and to run parallel to the DCN surface (Figures 9E,F). These characteristics were consistent with those of parallel fibers, the axons of granule cells.

While the numbers and proportions of shaft and spine terminals on the apical dendrites were very similar to each other, spine terminals were fewer in number and comprised a much lower proportion of the total number of terminals on the basal dendrites. From a total of 566 synapses on basal dendrites, only 65 (11%) were spine terminals. However, both types of terminals on the basal dendrites were strikingly large compared to their counterparts on the apical dendrites, with mean volumes of 10.58 ± 9.98 μm^3^ (mean ± SD) (*n* = 62) and 4.36 ± 6.37 μm^3^ (mean ± SD, *n* = 504) for the basal spine and shaft terminals, respectively. Spine terminal volumes ranged from 9.8 ± 10.64 μm^3^ in FC6 to 15. 32 ± 9.68 μm^3^ in FC7.

### Active zones and PSDs on axo-spinous synapses

**Figure 10:**
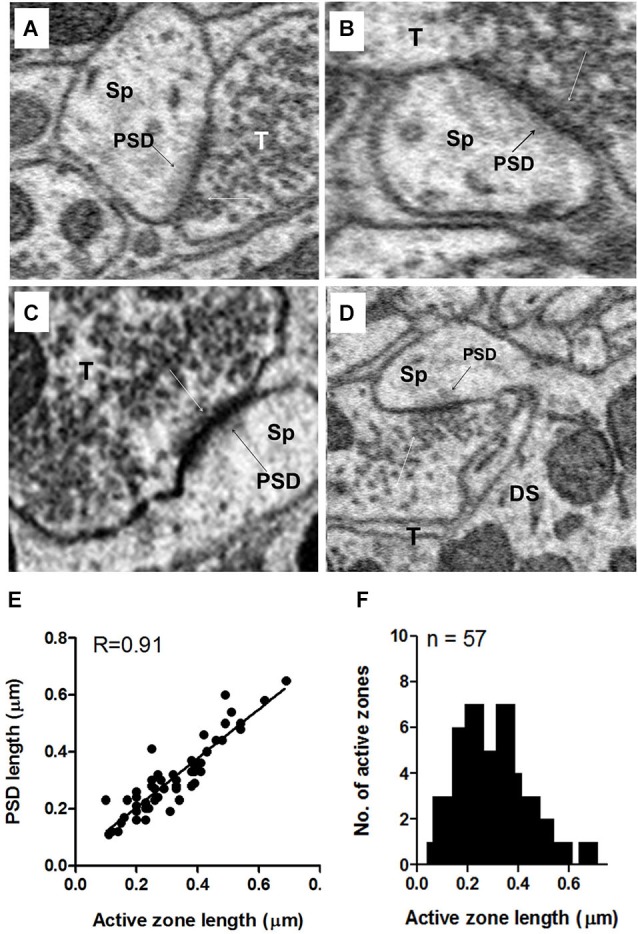
**Examples of axo-spinous synapses on apical dendrites showing active zones and postsynaptic densities (PSDs)**. **(A–D)** The axon terminal (T) abutting the spine (Sp) contained a single active zone with an associated cluster of docked vesicles (white arrows). The PSD appears as a dark shadow beneath the contact with the presynaptic terminal (T) of the spine. As in the terminals on the soma, the vesicle cluster associated with the active zone was an extension of a larger pool of vesicles that spread throughout and often filled much of the terminal. **(E)** Relationship between the active zone and PSD lengths of axo-spinous synapses. **(F)** Maximum length distribution of active zones of axo-spinous synapses. Data for **(E)** and **(F)** were obtained from a random sample of 57 synapses from the apical dendrites of three fusiform cells.

Unlike the terminals on somata (see above), the vast majority (80%) of terminals ending on apical dendritic spines had only a single active zone; less than 20% had two or three active zones. When more than one active zone was present in the same terminal, one active zone was adjacent to one spine while the other(s) was (were) adjacent to either a different spine or, less commonly, the shaft of the same or different dendrite. However, the terminals contacting the basal dendritic spines typically spread out to cover wide areas of the shaft, and these contained active zones adjacent to both spine and shaft, often with more active zones adjacent to the shaft than to the spine. As shown in Figure 10E, the lengths of the active zones and PSDs were highly correlated (*R* = 0.9, *p* < 0.0001). Active zone lengths varied across terminals on axospinous synapses, ranging from 0.095 to 0.66 μm, with a mean value of 0.30 μm + 0.13 (mean + S.D.) (Figure 10F). Terminals on spines were almost always flanked by a distinct PSD consisting of a darkly staining shadow zone just below the postsynaptic membrane that darkened the cytoplasm of the spine just below the point of synaptic contact (Figures 10A–D, black arrows). Thus, axospinous synapses were identifiable as type I (asymmetric) synapses. The terminals on shafts of both apical and basal dendrites were a mixture of symmetric and asymmetric synapses.

## Discussion

We have presented the results of a quantitative morphometric analysis and mapping of an array of synaptic features on DCN fusiform cells, concentrating on those that are most related to the strength of the synapse, in particular, the numbers, volumes and ultrastructure of synaptic endings, dendritic spines and postsynaptic densities. The goal of this analysis was to establish a normative baseline that would provide a foundation for future studies aiming to determine how manipulations of input and different pathological states affect synaptologies of fusiform cells. The use of SBFSEM provided the advantage of being able to perform such an analysis on neurons that were morphologically characterized and demonstrated to have all the key characteristics of fusiform cells that have been described in hamsters and other species commonly used in the laboratory (Blackstad et al., 1984; Schweitzer and Cant, 1985; Smith and Rhode, 1985; Rubio and Juiz, 2004).

### Regional differences in synapse size

Striking differences were found in the volumes of synapses contacting different regions of the cell. Terminals on the basal dendrites were generally much larger than those on the apical dendrites. The largest terminals were found on the spines of basal dendrites, while the smallest were on the spines of apical dendrites, particularly in the most superficial levels of the molecular layer. Terminal endings on the basal dendritic spines were more than six times larger than those on the apical spines, and those on the shafts of the basal dendrites were approximately twice as large as those on the shafts of apical dendrites. The terminals on the soma were approximately equal in volume to those on the shafts of apical dendrites but notably smaller than those on the basal dendrites.

The regional differences in terminal size provides some basis on which to infer the relative strengths of inputs to the different synaptic compartments of the cell. Synaptic strength is a function of multiple factors. One major factor for estimating synaptic strength is the probability of neurotransmitter release during axonal activation. Although measures of release probability were not within the scope of our study, there is evidence that release probability increases with the number of release sites, which correlates with the number and size of active zones within the presynaptic ending (Schikorski and Stevens, 1997). Thus, to determine indirectly whether larger synapses might have higher release probabilities, we compared both the number and sizes of active zones in soma as well as basal and apical dendritic terminals. Our results confirmed that the number of active zones increased linearly in all three of these compartments in proportion to the size of the synaptic ending. A similar relationship between number of active zones and terminal volume has been reported previously based on studies in neocortical neurons (Yeow and Peterson, 1991; Pierce and Mendell, 1993). These findings suggest that basal dendritic synapses are more powerful than those on the apical dendrites. If this is true, the association of small spines with large synapses observed on basal dendrites may seem paradoxical, given that small spines are usually correlated with factors (i.e., smaller terminals, fewer transmitter receptors, lower release probabilities) that are predictive of weaker synaptic strength (Harris and Stevens, 1988, 1989). However, unlike the terminals on apical dendritic spines, which were typically focused on spines, the large basal dendritic spine terminals typically spread out to contact large areas of the shaft and possessed multiple active zones adjacent to both the spine and shaft. Thus, the small basal dendritic spines may serve less of a role in spatial isolation of inputs than those on apical dendrites, but more to reinforce inputs to the shaft. A possible reason for this might be the need to boost the gain on input to basal dendrites, most of which comes from the auditory nerve or indirectly from the auditory nerve via inhibitory (vertical and D-stellate) or excitatory (T-stellate) cells that themselves receive direct excitatory input from the auditory nerve (Oertel et al., 1990, 2011; Doucet and Ryugo, 1997; Rubio and Juiz, 2004). Whether larger, more powerful synapses serve a particular physiological function (e.g., preserve more precise temporal information and focused tuning) or reflect the generally high rates of spontaneous and stimulus-elicited activity in the auditory nerve pathway is a topic for future investigations.

### Functional implications

The abundance of spines on the apical dendrites and relative paucity of spines on the basal dendrites is one of the key features that distinguish fusiform cells from other cell types in the DCN (Mugnaini et al., 1980a,b; Blackstad et al., 1984; Smith and Rhode, 1985). Spines are of special interest owing to their known role in receiving excitatory input and in mediating activity-dependent forms of plasticity such as long term potentiation (LTP) and long term depression (LTD). In the DCN, the main, if not exclusive, source of input to apical dendritic spines are the parallel fibers. Long term potentiation can be induced in fusiform cells by pairing depolarization of the soma with electrical stimulation of parallel fibers using high frequency pulses trains (HFS; Fujino and Oertel, 2003). Because parallel fibers provide the input to spines (Rubio and Wenthold, 1997), and NMDA receptors are found in spines (Petralia et al., 1994), apical spines are the likely sites of LTP-induction. In contrast, the basal dendrites have few spines (Ryugo and Willard, 1985) and little if any NMDA receptors (Bilak et al., 1996), and LTP is not induced when depolarization of the soma is paired with HFS of the auditory nerve, the main source of excitatory input to the basal dendrites.

At face value, these findings might be interpreted as implying that the basal dendrites lack the capacity for plasticity. However, numerous ultrastructural features observed in the present study suggest that synapses on basal dendrites may also be plastic. Although spines were generally scarce on all the basal dendrites of our fusiform cells, we noted a surprisingly dense distribution of spines on certain segments of those dendrites. In fact, the highest density of spines on the basal dendrites was comparable to the density of spines on some secondary branches of the apical dendrites. These basal dendritic spines were smaller than those on the apical dendrites, but their presence raises the possibility that some stretches of basal dendrites may possess the capacity for activity-dependent plasticity, albeit to a more limited degree than the apical dendrites. Another form of plasticity that may occur on the basal dendrites and other synaptic regions of the cell is dynamic remodeling and turnover of the presynaptic terminal. Sprouting of new synapses following injury is known to occur in the dorsal and ventral cochlear nuclei in response to injury (Bilak et al., 1996; Benson et al., 1997; Muly et al., 2002; Kim et al., 2004). However, imaging studies show that synapses, including their presynaptic terminals, are dynamic structures that undergo constant remodeling (Berghs and Roubos, 1996) and turnover even in the adult brain (Trachtenberg et al., 2002; Cavazos et al., 2003) as neurons connect, disconnect and reconnect with changes in activity. We observed a number of features that are suggestive of these forms of plasticity. First, while most terminals contained active zones, many completely lacked them, and even within the same terminal, active zones showed considerable variation in their size and distinctness, likely reflecting different stages of synapse formation. Additionally, almost all features that determine the strength of axo-dendritic and axo-somatic synapses and their influence on their postsynaptic targets (e.g., sizes of the terminal or number of active zones and mitochondria or the number of synapses connected by a common axon) varied along a more or less continuous gradient. These variations are consistent with the concept of the dynamically changing synapse whereby the precise morphological and ultrastructural characteristics of the pre-synaptic portion of the synapse vary with the state of activation. This would predict that these features would change with the level of acoustic activation. We plan future studies in which we will examine changes in the size and dimensions of features on the presynaptic side of the synapse with changes in levels of activation.

### Postsynaptic densities

Based on the presence or absence of PSDs, we differentiated synapses into the same two categories used by others, including asymmetric (Gray Type I synapses) which possess PSDs and symmetric (Gray Type II) which lack PSDs (see review of Klemann and Roubos, 2011). We quantified the relative numbers of these two types of synapses in each synaptic compartments of the cell. Our results showed that the different compartments could be distinguished by the proportions of each synaptic type. With very few exceptions, the synapses on dendritic spines were found to be asymmetrical synapses, while those on somata were mostly symmetric. The dendritic shafts (i.e., the segments of dendrites not covered with spines) were covered by a mix of symmetric and asymmetric synapses, with symmetric synapses composing 61% of apical shaft terminals and 43% of basal shaft terminals, the remainder being asymmetric synapses.

Many studies have adopted the concept that that asymmetric synapses are excitatory while symmetric synapses are inhibitory (see review of Klemann and Roubos, 2011). Based on this premise and the fact that most of the synapses we observed on dendritic spines of fusiform cells were asymmetric while those on the soma were predominantly symmetric, our results are not inconsistent with the view that axo-spinous synapses on fusiform cells are mostly excitatory while axo-somatic synapses are mostly inhibitory. This interpretation is also consistent with the previous finding that synapses on dendritic spines are glutamatergic while those on the soma are mostly a combination of GABAergic and Glycinergic endings (Rubio and Juiz, 2004). Further, our observation that synapses on dendritic shafts were a mixture of asymmetric and symmetric synapses implies a mixture of excitatory and inhibitory inputs, consistent with the findings of Rubio and Juiz (2004) that shaft synapses are a mix of glutamatergic and GABA-ergic/Glycinergic endings. In our sample, more than half of synapses on the basal dendritic shafts displayed PSDs and would thus be classified as excitatory according to the above scheme.

Some paradoxical relationships between asymmetric and symmetric synapses were observed in the present study suggesting that asymmetric and symmetric synapses may also be indicative of differences in function other than the polarity of the response they elicit. First, when axo-dendritic or axo-somatic terminals and their axons were traced through the image volume, numerous examples were found in which symmetric and asymmetric synapses (terminals) were linked to each other by the same axon or different branches of the same axon. In addition, when multiple active zones were found in a given synaptic terminal, some active zones were flanked on the postsynaptic side by distinct PSDs while others were not. An alternative interpretation of our results is that the presence or absence of PSD may reflect different degrees of synaptic stability, as recently discussed by Klemann and Roubos (2011). Accordingly, synapses with well-developed PSDs represent more established and more stable connections, while those without PSDs or with poorly developed PSDs are more transitional. If this interpretation is correct, our results would suggest that synapses on spines may be more uniformly stable from a structural standpoint to uphold their role in long term functional plasticity (Fujino and Oertel, 2003; Tzounopoulos et al., 2007), while those on the soma and dendritic shafts may be less secure but structurally more plastic as the endings compete with each other for access to the postsynaptic membrane. Future work examining the temporal dynamics of synapse formation on neurons using multiphoton imaging, as has been used to examine spine dynamics would provide an excellent approach to testing his hypothesis.

### Axo-somatic terminals on fusiform cells suggest a relatively small convergence ratio of inputs to fusiform cell somata

Our fusiform cell somata were contacted by an average number of 91 synapses per cell. The source of these inputs has not been determined in the hamster. However, in other species, at least seven sources of input to fusiform cell somata have been identified, including the auditory nerve (Kane, 1977), cartwheel and vertical cells of the DCN (Berrebi and Mugnaini, 1991; Rhode, 1999), at least one type of neuron (likely D and/or T-stellate cells) in the ventral cochlear nucleus (Oertel et al., 1990; Doucet and Ryugo, 1997), superior olivary complex (Kane, 1977), the nucleus of the lateral lemniscus and the inferior colliculus (Kane and Conlee, 1979). This suggests that the number of axons providing input to the soma from each of the seven sources of input to the fusiform cell soma might be 91 or roughly an average of 13 axons per source. However, when we traced axons retrogradely from their terminals over distances of a few micrometers, we found that each synapse was linked to an average of two other synapses by the same axon (i.e., 3 terminals/axon). This suggests that the number of axons providing input to the soma from each of the seven sources of input to the fusiform cell soma could average as few as 4 (13/3), and if the axons were traced further retrogradely and found to link even more terminals, the mean number of inputs from each source would likely be even smaller, possibly only 1 to 3.

### Dendritic spines on apical dendrites

The spines of fusiform cell apical dendrites displayed many of the same characteristics that have been found on the spines of other neuron types (e.g., cortical and hippocampal pyramidal neurons and cerebellar Purkinje cells). Spines on apical dendrites were distributed mainly on secondary and more distal branches of the dendrite and were most often contacted by a single synapse with a single active zone. A well-defined PSD was almost always found just beneath the postsynaptic membrane of the spine. Like the spines of cortical pyramidal neurons, the volumes of fusiform cell spines were variable but had a unimodal, asymmetrical distribution slightly skewed towards higher volumes.

Spines of fusiform cell apical dendrites also showed some characteristics that differed from those in the neocortex, hippocampus and cerebellum. Few of our spines displayed the classic mushroom shapes with long narrow necks and large bulbous heads that are observed on neurons in the neocortex (Harris et al., 1992; Bourne and Harris, 2007). Most of the spines on fusiform cells were stubby, with short necks that were poorly differentiated from the heads; consequently, it was not possible to obtain separate measures of spine neck and head volumes. Unlike spines on neocortical cell or hippocampal pyramidal cell dendrites (e.g., Konur et al., 2003), spines on fusiform cells dendrites were distributed irregularly, with dendritic segments of high spine density alternating with segments in which spines were either sparse or absent. These differences suggest that fusiform cells are uniquely customized for a form of processing that may not be shared by these higher order structures.

### Astrocytic processes surrounding the fusiform cell soma

For many years, it has been known that astrocytes are intimately associated with synaptic terminals and are often found contacting the cell surface and its dendrites (Araque et al., 1999). Our results show that the astocytic processes occupy a major share of the soma surface, forming a sheath around the cell, except where axosomatic and axospinous terminals are present. In many instances the astrocytic processes formed several layers around the soma, analogous to the wrapping of myelin around an axon. There is a growing evidence that astrocytes play a role in determining where on the cell surface synapses contact neurons (Bolton and Eroglu, 2009; Eroglu and Barres, 2010; Kucukdereli et al., 2011). If this is true, then the fact that so much of the fusiform cell somatic surface was covered with astrocytic processes suggests that these glia cells have additional capacity for adding and/or removing synapses to the fusiform cell soma or changing the size of existing synapses. Thus, because the area covered by astrocytic processes is inversely related to the area covered by synapses, astrocytes may control the number, sizes and distribution of synapses and hence the relative weight of their influence on the postsynaptic cell.

## Conflict of interest statement

The authors declare that the research was conducted in the absence of any commercial or financial relationships that could be construed as a potential conflict of interest.
